# Resilience through resistance: the role of worker agency in navigating algorithmic control

**DOI:** 10.3389/frai.2025.1600044

**Published:** 2026-01-16

**Authors:** Morgan Williams, Uma Rani

**Affiliations:** Department of Research, International Labour Organization (ILO), Geneva, Switzerland

**Keywords:** algorithmic management, control resistance relationship, digital labor platforms, resilience, worker agency, worker contestation, worker resistance

## Abstract

**Introduction:**

The business model of multi-sided digital labor platforms relies on maintaining a balance between workers and customers or clients to sustain operations. These platforms initially leveraged venture capital to attract workers by providing them with incentives and the promise of flexibility, creating lock-in effects to consolidate their market power and enable monopolistic practices. As platforms mature, they increasingly implement algorithmic management and control mechanisms, such as rating systems, which restrict worker autonomy, access to work and flexibility. Despite limited bargaining power, workers have developed both individual and collective strategies to counteract these algorithmic restrictions.

**Methods:**

This article employs a structured synthesis, drawing on existing academic literature as well as surveys conducted by the International Labour Office (ILO) between 2017 and 2023, to examine how platform workers utilize a combination of informal and formal forms of resistance to build resilience against algorithmic disruptions.

**Results:**

The analysis covers different sectors (freelance and microtask work, taxi and delivery services, and domestic work and beauty care platforms) offering insights into the changing dynamics of worker agency on platforms, which have enabled resilience-building among workers on digital labour platforms. In the face of significant barriers to carrying out formal acts of resistance, workers on digital labour platforms often turn to informal acts of resistance, often mediated by social media, to adapt to changes in the platforms’ algorithms and maintain their well-being.

**Discussion:**

Platform workers increasingly have a diverse array of tools to exercise their agency physically and virtually. However, the process of establishing resilience in such conditions is often not straight-forward. As platforms counteract workers’ acts of resistance, workers must continue to develop new and innovative strategies to strengthen their resilience. Such a complex and nuanced landscape merits continued research and analysis.

## 1 Introduction

Advancements in mobile technologies and cloud computing paved the way for digital labor platforms, (hereafter, platforms), to facilitate on-demand exchanges of services that take place between workers and customers or clients, and the workers on these platforms are often classified as self-employed or independent contractors (ILO, 2021). Over the past decade, platforms have expanded across a diverse range of sectors, becoming an increasingly common feature of the modern economy and society. While platforms provide opportunities for marginalized workers, including women and migrant workers, their classification as self-employed workers or independent contractors does not allow them to access standard labor and social protections and shifts the responsibility of investing in assets and risks to workers (ILO, 2021). Further, the use of algorithms by the platforms for managing work processes impacts their access to work and income stability.

While ubiquitous on platforms, algorithmic control is not an inherent feature, but a deliberate strategic choice made by platforms to maximize workers’ output and unpaid labor (Dhar and Thuppilikkat, 2022). The evolution of managerial control on digital labor platforms resembles a Trojan horse strategy, wherein platforms initially present themselves as flexible alternatives to traditional employment, only to later implement algorithmic control mechanisms once market dominance is established. Extensive empirical evidence indicates that workers do not passively accept this widening power imbalance. Instead, they engage in a continuous struggle against platforms seeking to control the work process (Gandini, 2018; Joyce and Stuart, 2021; Bessa et al., 2022).

The process through which workers exercise their agency to rebalance power and protect their well-being – financial and health well-being—can be conceptualized as a process of resilience building. Resilience, a multi-disciplinary concept, varies in interpretation across sectors. Anwar and Graham (2020) pioneered research on how platform workers assert agency through resilience, reworking and resistance to improve their working conditions. However, their conceptualization does not recognize the inter-connected and dynamic nature of resilience, which may involve acts of resistance as a means to achieve it. We propose a new model of resilience on digital labor platforms by arguing that once workers are locked into platforms, the implementation of algorithms acts as a shock to their well-being, which is then mitigated by informal and formal acts of resistance designed to circumvent, mislead, or even, fundamentally alter platform polices. This model presents a more nuanced, process-oriented view of resilience, recognizing its dynamic nature and the need for diverse methods and responses to effectively restore well-being in the face of algorithmic control.

This article draws on a decade of academic literature as well as surveys and research conducted by the International Labour Office (ILO) to demonstrate how platform workers combine informal and formal acts of resistance in response to the implementation of algorithmic control. In doing so, we expand and cover a diverse range of geographic boundaries and sectors (taxi and delivery, freelance and microtask, domestic and beauty work services), thereby enabling comparability. We also provide a focused analysis of how workers exercise resistance to algorithms, highlighting the paradox where workers depend on technology for their labor, are governed by it, and simultaneously leverage it as a tool for organization and resistance. The following sections conceptualize managerial control, resilience, and resistance before presenting examples of informal and formal resistance methods that demonstrate the use of worker agency in a sector where workers otherwise possess limited structural power.

## 2 Materials and methods

### 2.1 The evolution of managerial control on digital labor platforms

**Figure 1:**
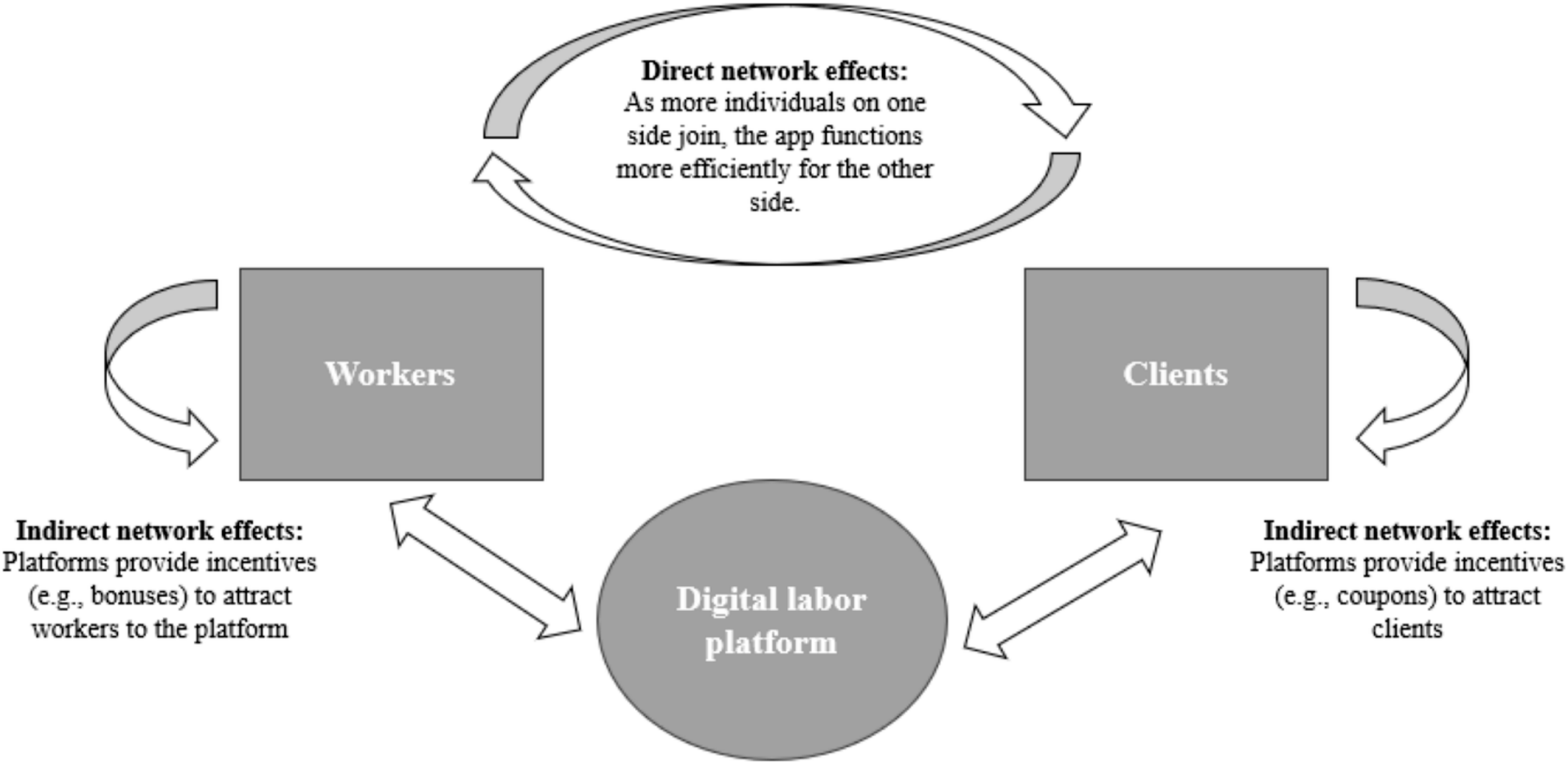
Simulating network effects on a two-sided platform. Source: Adapted based on Srinivasan (2021). The figure illustrates how digital labor platforms operate as an intermediary between workers and clients, as well as how the generation of direct and indirect network effects is critical to creating an appropriate balance between workers and clients on the platform.

The business model of multi-sided platforms requires a sufficient number of users on each side for the platform to function effectively, without creating delays or inefficiencies for users on either side. As the user-base grows on one side (e.g., workers), the platform becomes more valuable to users on the other side(s) (e.g., clients), which in turn, establishes positive feedback loops to stimulate growth (Evans and Schmalensee, 2008; Li et al., 2010) (Figure 1). The enhanced pressure to achieve these network effects early on comes from the possibility of securing a space in the winner-takes-all market, where only a few platforms survive (Kenney and Zysman, 2016). Winners, as a result, act as monopolists, centralizing their power to maximize their gains through adjustments to their business models or acquisitions of other platforms to expand functionalities (ILO, 2021). This critical role of network effects for effective functioning, coupled with the promise of market dominance, significantly motivates platforms to adopt pricing and non-pricing strategies that attract users across all sides (ILO, 2021).

To stimulate network effects, platforms offer preferential services to attract workers. In both online web-based platforms and location-based platforms, job flexibility is a primary motivation for engaging in platform work (ILO, 2021) (Figure 1). Platforms often promise workers that they provide flexibility over their work schedules, and the ability to work from any geographic location. This flexibility is particularly appealing to women, enabling them to balance platform work with their household and care responsibilities (ILO, 2021; Rani et al., 2022). Moreover, workers report that platforms address the inability to find other suitable employment by creating new opportunities and eliminating tedious entry barriers for vulnerable populations facing labor market discrimination, such as migrant workers and those with disabilities (Berg et al., 2018; ILO, 2021). Migrant workers, for instance, utilize platforms to overcome barriers relating to language proficiency, unrecognized credentials, and discrimination, that often hinder labor market integration (ILO, 2021).

From the platform’s perspective, online web-based platforms have expanded employers’ access to a global workforce. This has extended the reach of globalization by establishing skill arbitrage, enabling firms in the global North to access workers with lower reservation wages in the global South (Rani et al., 2025). The expansion opportunities on online web-based platforms reflect, to some degree, the exploitation of the often invisible human labor necessary to train artificial intelligence (AI) systems to function (Crawford, 2021; Rani et al., 2025, 2026). Some argue that online freelance workers in the global South, in particular, might be able to benefit by setting their prices and conditions higher than possible in their local labor markets (Vandaele, 2021), which is questionable and there is no evidence to prove this claim. Skill arbitrage often relies on workers possessing specialized skills that grant them with some degree of bargaining power, which may not be generally true in many developing countries (Rani et al., 2025, 2026). While workers on location-based platforms may not have the same degree of bargaining power as workers on online web-based platforms, survey evidence suggests that hourly earnings for app-based workers in the taxi and delivery sectors outpace the traditional sectors in several countries in the global South (ILO, 2021). This disparity can be partly attributed to platforms disrupting traditional local labor markets by offering low-cost services to customers while simultaneously providing bonuses and incentives to workers (ILO, 2021).

Platforms offer a variety of monetary and non-monetary incentives to attract and retain workers. Both online web-based platforms and location-based platforms employ fixed and contingent bonuses to compete for workers against other platforms and the traditional labor market (Chen et al., 2025; ILO, 2021). The effectiveness of these bonuses is dependent on the country or city, and the market conditions, with fixed bonuses being more effective in competitive markets and contingent bonuses in high-demand environments. Sign-up bonuses and referring programs are common strategies to attract drivers or workers to the platform (ILO, 2021, 2024a, 2024b). A notable strategy that has been popularized by on-demand taxi platforms to retain workers is surge pricing, which uses real-time data to balance labor supply and demand by rewarding workers who respond to requests in high-demand areas (Castillo, 2025). Monetary and non-monetary incentives, conversely, often involve gamification tactics, which are designed to influence workers’ behavior through scores or badges or additional payments for reaching specific targets that signal success or establish a hierarchy (ILO, 2021). Both surge pricing and gamification tactics highlight the critical role algorithms play in shaping workers’ behavior to maximize productivity.

The provision of generous incentives raises questions about their financial sustainability. The pressure to establish networks effects often requires platforms to subsidize one side with the profits from another (Evans and Schmalensee, 2008). However, these subsidies frequently fall short, leading many platforms to operate at a loss. Venture capital has been instrumental in bridging this funding gap, with the expectation that platforms will achieve market concentration and yield substantial long-term gains (ILO, 2021). By enabling platforms to operate at a loss, venture capitalists have contributed to the mass disruption of traditional sectors that cannot compete with the bonuses offered to workers and the low costs for consumers, irrespective of their revenues. Some taxi platforms have utilized venture capital to subsidize drivers and consumers, consolidating “artificial market power” in the taxi market, at prices unfeasible for asset-heavy taxi companies to charge (Kenney and Zysman, 2016; Horan, 2019, p. 5; ILO, 2021).

As positive feedback loops continue, platforms achieve critical mass on all sides, and as market power consolidates, the need to attract users through incentives diminishes. Once platforms have established lock-in effects, such as dependence on the platform to access tasks or rides or projects and restructure the market such that workers are unlikely to leave the platform for local labor market or traditional industries, then they begin altering changes to their policies as well as to the algorithms. This signifies a power shift from workers to platforms, often accompanied by the introduction of more intensive algorithmic control mechanisms and the reduction of previously provided protections including bonuses and incentives. Platforms exploit this power to effectively “squeeze workers’ labor and time” (Dhar and Thuppilikkat, 2022, p. 273). For instance, female workers in beauty parlors in India, were initially drawn to platforms by the idea that technology would be more fair and less biased. However, once the platforms had achieved sufficient user lock-in and workers dependent on it, they eliminated the worker flexibility and intensified control through algorithmic management practices (Dhar and Thuppilikkat, 2022). This left workers, who had exited traditional beauty parlors, with little choice but to submit to algorithmic control.

### 2.2 Effects of algorithmic control on worker agency

A platform’s capacity to gather, harness, and leverage data is fundamental to its ability to extract value, boost productivity, and to further consolidate market power. Consequently, the impact on platform workers’ working conditions depends on how platforms use and deploy the data. Platforms feed data into algorithms that automate or semi-automate core management functions—direction, evaluation, and discipline—a process known as algorithmic management (Kellogg et al., 2020; ILO, 2021). Similarly, Moore and Joyce (2020) explain that despite the differences, platforms employ a range of technical and organizational management methods referred to as platform management methods (PMM)^1^ for varying degrees of work organization and control (Cini, 2022; Joyce and Stuart, 2021). While these tools are designed to enhance efficiency by streamlining decision-making and coordination, the increasing reliance on algorithmic decision-making raises concerns about diminished worker agency and data privacy (Kellogg et al., 2020; Moore and Joyce, 2020; Joyce and Stuart, 2021; Cini, 2022). The theory of surveillance capitalism posits that algorithms require the continuous extraction of vast amounts of data, behavioral data or information (Zuboff, 2019). Platform workers, in particular, are susceptible to data extraction across all aspects of their work process (e.g., location data, performance data, behavioral data), which can often be exploited by managers for profit and for intensified control over workers.

Platforms implement a combination of PMM to suit their strategies, resulting in variations in algorithmic management methods both within and between sectors. For example, online freelancers generally have greater agency over employment terms and task selection, whereas delivery drivers are often automatically matched with tasks (Vandaele, 2021). Platforms use *algorithmic direction* to inform workers about tasks to be performed, in what time period, in a specific sequence, or with a certain degree of accuracy (Kellogg et al., 2020). These directions can be explicit, such as matching a client to a worker, or disguised as gamified notifications or behavioral nudges designed to influence worker behavior. These nudges might offer monetary or non-monetary incentives to complete a certain number of tasks or work in specific locations (e.g., surge pricing) (Kellogg et al., 2020; Kerényi, 2021). Such personalized notifications rely on machine learning algorithms that identify patterns in worker data to make recommendations (Kellogg et al., 2020). For instance, following the 2018 redesign of the Uber application in the United States, drivers were introduced to new features, such as individualized challenges and badges to coerce them into accepting additional work to meet demand (Vasudevan and Chan, 2022). These psychologically engineered games cultivate obsessive behaviors that compel workers to undertake challenges (Kim and Werbach, 2016, p. 164) and remain engaged on platforms. Not only do these nudges squeeze labor, but they also impact work-life balance, often requiring workers to work during asocial hours and leading to frustration when workers lack the agency to override recommendations. Additionally, algorithms have been observed to withhold ride assignments as drivers approach bonus targets, making attainment difficult. This algorithmic manipulation, hindering drivers from reaching their goals, has been documented in other countries as well (Rosenblat and Stark, 2016). Nearly half (43 percent) of app-based drivers reported increased difficulty qualifying for bonuses due to constantly shifting platform requirements (Rani et al., 2022). This also has significant implications for their incomes and livelihoods, especially since such bonuses constitute a substantial portion of their earnings. The suppression of worker agency leaves workers with a sense of powerlessness against an anonymous, faceless manager.

*Algorithmic evaluation* and *algorithmic discipline* operate in tandem as a form of algorithmic control, commonly introduced only after workers are locked into the platform. The integration of one-sided and two-sided rating systems, often outsourced to customers or clients, lies at the core of performance management. Workers with high customer ratings can gain greater access to work, while those with lower ratings may be penalized or even automatically deactivated without a clear recourse mechanism (De Stefano and Wouters, 2019; ILO, 2021; Joyce and Stuart, 2021). Among workers on location-based platforms, 19 percent of taxi drivers and 15 percent of delivery workers have experienced account deactivation (ILO, 2021). The consequences of these ratings are often opaque, unpredictable, and influenced by factors beyond workers’ control, such as delays, traffic, or customer dishonesty. For example, customers may falsely manipulate rankings on online web-based platforms by rejecting work without convincing reasons, and on location-based platforms by giving low ratings for malicious reasons, such as to avoid paying for a trip (ILO, 2021). As many as 86 percent of workers on microtask platforms and 34 percent of workers on freelance platforms reported their work being rejected, with many suggesting that the rejections were unjustifiable (ILO, 2021). The threat is exacerbated for new entrants and workers whose ratings reflect implicit and explicit biases that limit their access to work and, by extension, their earnings (Rani et al., 2022). The risks of bias, harassment, and discrimination are compounded for minority workers with intersecting identities –such as migrant women – who face multiple forms of vulnerability (Hester et al., 2020). However, sometimes top-rated freelancers on major platforms are allowed to remove or hide their low ratings (Rani et al., 2023). The combination of ratings and the lack of transparency or predictability of their consequences imposes significant pressure on workers to accept mistreatment (including physical and psychological abuse), which in turn, puts their physical and mental health at risk (Huws et al., 2017; Joyce and Stuart, 2021). Even in cases where harassment and discrimination are not primary concerns, many workers reported being unable to decline requests without facing implications for their ratings, resulting in lost bonuses, reduced access to work, and even deactivation (ILO, 2021). Consequently, the delayed introduction of algorithmic evaluation and discipline undermines autonomy and erodes the promise of flexibility that initially attracted workers to platforms. However, rating systems are not essential and should not be accepted as a permanent feature on platforms. Wolt (2024) a Finnish food delivery pltform, for instance, ranks and recommends restaurants based on consumer’s location, opening hours, time of day, and purchasing behavior, rather than relying on customer rankings that can be used to punish workers.

### 2.3 Conceptualizing resilience in the context of digital labor platforms

The cross-disciplinary study of resilience offers a deeper understanding of how individuals, communities, systems, and organizations adapt to challenges (Herrman et al., 2011; Nisioti et al., 2023; Southwick et al., 2014). Much of the early literature on resilience originated in medical fields, such as psychology and neuroscience, focuses on the short- and long-term consequences of stress (Southwick et al., 2014). For instance, the American Psychological Association, defines resilience as, “the process of adapting well in the face of adversity, trauma, tragedy threats, or even significant sources of threat” (Southwick et al., 2014, p. 259). While these disciplines approached resilience from an individualistic perspective, crises affecting large populations—natural disasters, climate change, the Great Recession, and the COVID-19 pandemic—have paved the way for a broader collective and societal conceptualization of resilience.

Resilience-building approaches in labor economics and international development have laid the groundwork for understanding resilience on platforms. Labor market resilience is typically understood in relation to macro-economic recovery from endogenous and exogenous shocks, particularly economic recessions. While labor market resilience is often monitored and evaluated using macroeconomic indicators such as employment in the literature, researchers acknowledge the micro-economic mechanisms that facilitate recovery. Social dialogue and government-led active labor-market policies are crucial for establishing adaptability in labor markets (Jara and Faggian, 2018; Hijzen et al., 2017; Moro et al., 2021; Scarpetta, 2018). However, these traditional systems of social dialogue and government policy are, however, designed for standard employment relationships and may not be appropriate for addressing work on platforms (Scarpetta, 2018).

In the context of new forms of work such as platform work, resilience aligns more closely with the international development discipline. A modern definition of development resilience encompasses the transformation of individual and collective well-being to avoid psychological stressors, such as low standards of living, that accompany environmental and societal shocks (Barrett and Constas, 2014; Béné et al., 2014; Nisioti et al., 2023). International development places human well-being at the center, thereby establishing a normative foundation absent from disciplines that rely on descriptive statistics as benchmarks for resilience (Barrett and Constas, 2014). For this reason, this theory is appropriate for examining the strategies platform workers individually and collectively use to adapt to changes in their work environment to maintain or improve their well-being.

Anwar and Graham (2020) were the first to apply the concept of resilience to platforms, examining how platform workers exercise agency through “resilience,” “reworking,” and “resistance” to improve their working conditions, a theory originally proposed by Katz (2004) in the context of political and economic transformations in the world of work that influence their environments. The authors adopt a relatively muted definition of resilience, as “small acts of ‘getting by’ or coping with everyday realities without necessarily changing existing social relations” (Anwar and Graham, 2020, p. 1273). According to them, platform workers demonstrate resilience by sharing accounts, posting advice on social media groups, or buying reviews, among other examples, highlighting the everyday nature of these acts of agency (Anwar and Graham, 2020). Resistance, on the other hand, directly targets clients to “confront and redress historically and geographically specific conditions of oppression and exploitation” (Anwar and Graham, 2020, p. 1272). These definitions place the three terms at the same level and create only a minor distinction between resilience and resistance that can be difficult to disentangle or operationalize.

**Figure 2:**
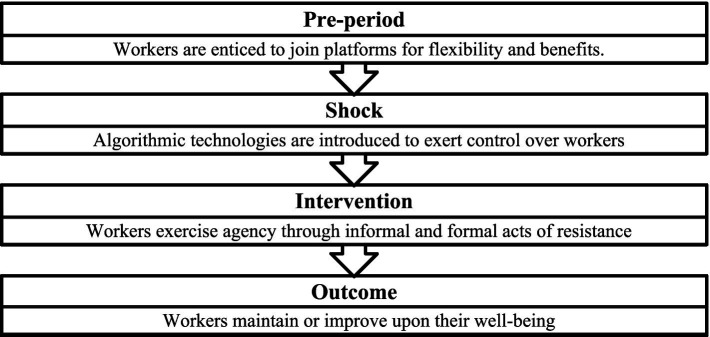
The trajectory of resilience-building on digital labor platforms. Source: Authors’ own illustration. The figure illustrates the process by which individuals and collective groups build resilience to algorithmic changes on digital labor platforms.

Instead of treating resilience, reworking and resistance as mutually reinforcing practices, this article advances the conceptualization of resilience on platforms by framing resistance as an *intervention* workers use to build resilience (Figure 2). In this context, the algorithmic control designed by platforms to limit worker agency is not absolute but is actively contested through both formal and informal acts of resistance (Vandaele, 2021). These interventions emerge in response to the introduction or changing strategies of algorithms and, in turn, function as mechanisms for reestablishing the flexibility and autonomy that initially characterized platform work.

### 2.4 Exercising worker agency through resistance

The inherent conflict between managerial control and worker resistance, a cornerstone of labor process theory, remains highly relevant in the contemporary platform economy (Gandini, 2018; Joyce and Stuart, 2021; Bessa et al., 2022). While algorithms have reshaped how managers exert control, they have not rendered workers immune to resistance. Joyce and Stuart (2021) caution against the “panacea fallacy” emphasizing that increased control inevitably fosters an increased drive to resist (Joyce and Stuart, 2021, p. 163). In this dynamic environment, workers demonstrate remarkable creativity, innovating both individual and collective forms of organization across diverse geographies. They employ both well-established and innovative methods of *formal* and *informal* acts of resistance.

Drawing on Scott (1985) and Scott (1990) theory of “hidden transcripts,” Anwar and Graham (2020) demonstrate some of the subversive ways platform workers exercise agency outside direct employer supervision. “Hidden transcripts” are an example of *informal* resistance, discrete initiatives allowing workers to exercise agency and improve their working conditions without provoking retaliation (Anwar and Graham, 2020; Joyce and Stuart, 2021; Dhar and Thuppilikkat, 2022; Tandon and Sekharan, 2022). Consequently, workers challenge employer dominance through a broad spectrum of activities, ranging from foot dragging and false compliance to more extreme acts like arson or sabotage (Ackroyd and Thompson, 1999; Cioce et al., 2024; Crowley et al., 2014; Hodson, 1995; Moyo, 2018; Scott, 1985). These actions exist on a continuum, from simple rule-breaking to serious criminal activity (Joyce and Stuart, 2021). Silver (1995), in her conceptualization of labor unrest (i.e., how workers intentionally resist to the commodification of labor), argues that “hidden transcripts” become labor unrest when they “reach a widespread and pathological level” (p. 20). This inclusion legitimizes the critical role of “weapons of the weak” in rebalancing power dynamics in the face of managerial control.

While Scott (1985) coined “weapons of the weak” in the 1980s, digital transformation has significantly expanded the methods of resistance available to workers (Dasgupta et al., 2025a). In the context of globally dispersed digital labor platforms, informal acts of resistance frequently manifest digitally, either in the form of sharing tips and tricks on social media groups (ILO, 2021; Vandaele, 2021), purchasing good ratings (Anwar and Graham, 2020), strategically ignoring algorithmic nudges (Kellogg et al., 2020), or negotiating wages outside of platforms (Dhar and Thuppilikkat, 2022). These actions highlight a paradox: even as algorithms disrupt the work environment, workers leverage other technologies to cultivate resilience and collectively regain control.

*Formal acts* of resistance are visible organized efforts aimed at achieving systemic policy changes in worker rights and legal recognition. Workers can leverage organized labor unrest—through protests, strikes, and coordinated logoffs –in both physical and online spaces to draw widespread public attention to their grievances (Bessa et al., 2022). These two forms of resistance are not isolated; informal actions often establish priorities and impetus for mobilizing formal resistance activities, which hold the potential for long-lasting structural change.

Despite ILO Conventions No. 87 and No. 88 affirming the right of all workers, including platform workers, to organize and engage in collective bargaining, the role of traditional trade unions in the platform economy remains limited. The self-employed and geographically dispersed nature of platform contributes to this challenge, with ILO country and global surveys indicating low union membership: only 5 percent of microtask workers, 1 percent of freelance workers, and less than 3 percent of app-based taxi drivers (ILO, 2021). Nevertheless, platform workers are finding innovative ways to collectively organize, thereby asserting their agency and achieving a level of structural power that surpasses individual informal acts of resistance (Vandaele, 2021).

*Lessons from informal worker organizing*: Historically, informal workers have faced barriers to organizing similar to those encountered by platform workers. Precarious living and working arrangements, the absence of traditional legal protections, the individualized nature of their work, and the opportunity cost of organizing, all hinder collective action (Bonner and Spooner, 2011). While some informal workers integrate into formal trade unions or establish their own informal worker associations, flexible alternative forums for advocacy are also available through cooperatives and non-governmental organizations (NGOs), including national and international networks (Bonner and Spooner, 2011; Rani and Sen, 2018). The Self-Employed Women’s Association (SEWA) in India, representing over a million women in the informal sector, exemplifies how grassroots associations can effectively organize informal workers to raise awareness of rights, facilitate access to social protection, and mobilize collective action, such as strikes and sit-ins, to advocate for policy reform (Bonner and Spooner, 2011; Rani and Sen, 2018).

The effectiveness of worker resistance fundamentally hinges on the degree of *structural power* wielded by platform workers. Structural power comprises two main dimensions: *workplace bargaining power*, defined by workers’ capacity to disrupt business operations through resistance, and *market power*, characterized by high demand for their labor (Silver, 2003). Ostensibly, platform workers exhibit limited structural power compared to the traditional sector on both fronts. The high global or local labor supply and scarcity of tasks restrict their ability to disrupt business, and their skills are often not sufficiently scarce (Vandaele, 2021; Dhar and Thuppilikkat, 2022). However, the extent of structural power varies significantly across sectors and is shaped by platform policies. Online freelance workers, particularly those with specialized skills, often possess a distinct advantage in market power, leveraging their expertise for greater bargaining leverage. Yet, their global dispersion and fierce competition with an ever-increasing global labor supply often makes it difficult to collectively organize (Rani et al., 2026). In contrast, taxi and delivery workers demonstrate strong workplace bargaining power due to their capacity to disrupt local transportation and distribution networks. This inherent leverage, Vandaele (2021) argues helps explain the higher prevalence of collective agreements in location-based platforms. For platforms without these sector-specific advantages, strengthening structural power is primarily achievable through collective action that generates external pressure on platforms.

### 2.5 Methodology

Within the complex techno-social power dynamics outlined in the previous sections, this article aims to explore resilience-building strategies among platform workers through the following research questions:

- RQ1: How do platform workers exercise agency through informal and formal acts of resistance to adapt to changes in algorithmic control?
- RQ2: How do informal and formal acts of resistance vary across different types of platforms?

This article addresses its central questions by drawing on primary surveys conducted by and in collaboration with the ILO between 2017 and 2023, encompassing both country-level surveys of location-based platforms and global surveys of online platforms. The surveys were conducted in the following platform sectors—online freelance and microtask platforms, taxi and delivery platforms, domestic work and beauty platforms.

The country level surveys for location-based platforms were meticulously implemented in collaboration with local researchers and institutes. The selection of these countries was predicated on the imperative to achieve regional diversity, specifically requiring geographical representation across the Global South. They were further chosen for their high platform penetration at the time of the survey and the presence of varied institutional and regulatory environments. To ensure cultural and linguistic relevance, questionnaires underwent adaptation to local contexts and translation into local languages, a process guided by consultation with in-country researchers. A pilot test preceded the main data collection in each country to identify and resolve any potential issues. Interviews were administered using Computer-Assisted Personal Interviewing (CAPI) on mobile devices, such as cell phones and tablets, benefiting from built-in validation rules to ensure data quality. Respondents received a fixed, country-specific payment to compensate for their time, with each survey averaging approximately 40 minutes to complete.

Given the absence of official statistics on platform workers, a traditional random sampling frame was not feasible. Therefore, the primary objective was to achieve a sample as representative as possible of the target population, defined as individuals aged 18 or older with at least 3 months of experience in the relevant sector to ensure informed responses. Recruitment strategies were tailored to each sector: taxi drivers were targeted at locations such as gas stations, office complexes, shopping malls, airports, railway stations, platform company support offices, and taxi stands. Delivery workers were primarily recruited near restaurants, shopping malls, and other common waiting areas. For beauty workers, recruitment involved Facebook advertisements, alongside lists of consented workers provided by some platforms. Healthcare respondents were initially reached via personal contacts and referrals, with subsequent participants recruited using a snowball sampling method. While diverse recruitment strategies were essential for accessing workers who are otherwise difficult to reach, they may introduce selection bias. This could limit generalizability of findings, such as through the oversampling of drivers and riders in specific geographic areas, or of beauty workers who are more likely to use or are active on Facebook.

The 2017 global microtask worker survey and the 2019–2020 freelance survey were conducted with the assistance of SoundRocket, a social science survey research firm. The microtask survey was disseminated as a paid task across five different platforms, with open participation except for Amazon Mechanical Turk (MTurk), where it specifically targeted workers from India and the United States. To ensure sample diversity, the survey was posted in small batches at various times throughout the day, allowing workers to self-select their participation. For the freelance survey, a multi-faceted recruitment strategy was adopted after evaluating several models. The final approach involved direct recruitment on the freelance platform (90 percent), identification of workers through other digital platforms like MTurk (8 percent) and targeting individuals via online advertisements (2 percent). Similar to the sector-specific surveys discussed above, these strategies facilitated the access to hard-to-reach workers or populations. However, they may have resulted in the oversampling of workers in certain geographic areas and those with higher exposure to targeted online advertisements. All participants who completed the freelance questionnaire received compensation for their time, with the survey taking approximately 60 min to complete.

The data derived from these surveys were meticulously disaggregated by gender, country, sector, and platform type (online or location-based). The data were subsequently classified into two overarching themes of informal and formal resistance, with further sub-themes based on specific worker strategies. Where feasible, these findings were supplemented with academic articles, which were selected based on their methodological approach (i.e., primary surveys and case studies), all rigorously assessed for methodological quality. This evidence represents a structured synthesis which provides a comprehensive understanding of the varied forms of worker resistance across different contexts, highlighting both the commonalities and distinctions in strategies employed within the platform economy. Table 1 provides a comprehensive list of analyzed articles and their methodologies.

**Table 1:** ILO and non-ILO research on resistance in the platform economy.

Source	Country or regional coverage	Sector	Method
Altenried (2020)	Global	Microtask	Secondary research
Anwar and Graham (2020)	South Africa, Kenya, Nigeria, Ghana, Uganda	Freelance	In-depth interviews with 65 workers
Berg et al. (2018)*	75 countries	Microtask	2015 survey (n = 1,167) and 2017 survey (n = 2,350)
Bessa et al. (2022)*	Global	Taxi; Delivery	Leeds Index of Platform Labour Protest
Chen et al. (2025)	N/A	General	Game theory model
Cini (2022)	Global	Microtask	Systematic collection of scientific publications and policy reports
Dhar and Thuppilikkat (2022)	India	Domestic work and beauty services	Case study of workers at one company
Ferrari and Graham (2021)	Europe, Africa, Asia	Mixed	One-on-one interviews, surveys, focus groups, and action research workshops
Hadwiger (2022)*	Argentina, Chile, China, Ghana, India, Indonesia, Kenya, Lebanon, Mexico, Morrocco, Ukraine	Microtask; Taxi; Delivery	Global surveys and country-specific (China and Ukraine) surveys of online-web-based platforms; App-based and traditional surveys of location-based sectors
Heiland (2020)	Global	General	Secondary research
ILO (2021)*	Argentina, Chile, China, Ghana, India, Indonesia, Kenya, Lebanon, Mexico, Morrocco, Ukraine	Freelance and contest-based; Competitive programming; Microtask; Taxi; Delivery	Global surveys and country-specific (China and Ukraine) surveys of online-web-based platforms; App-based and traditional surveys of location-based sectors
ILO (2024a)*	Kenya	Domestic work and beauty services; Healthcare; Tutors; Personal services; Taxi; Delivery; Freelance; Microtask; Marketplace; AI-BPO	Country-specific survey across a range of platform and traditional sectors
ILO (2024b)*	Uganda	Taxi; Delivery; Freelance; Marketplace	Country-specific survey across a range of platform and traditional sectors
Möhlmann and Zalmanson (2017)	United Kingdom (London); United States (New York)	Taxi	Interviews with drivers and analysis of blog posts
Rani et al. (2023)*	57 countries in Asia, Africa, Arab States, transition countries of Central and Eastern Europe, Latin America	Freelance and contest-based; Competitive programming; Microtask	Surveys of 1,231 workers in developing countries
Rani et al. (2025)*	Global	Freelance	Surveys and interviews with freelance workers in global North and South
Rani et al. (2026)*	India, Kenya	Microtask; AI-BPO	Surveys and interviews with microtask workers
Salehi et al. (2015)	Global	Microtask	Ethnographic fieldwork of the MTurk community
Tandon and Sekharan (2022)	India	Domestic work and beauty services	Case study of workers at one company
Tassinari and Maccarrone (2020)	Italy; United Kingdom	Delivery	Case study of two cases of mobilization
Qadri and D’Ignazio (2022)	Indonesia (Jakarta)	Taxi	Ethnography and interviews with 6 driver communities
Woodcock (2021)	Global	Taxi; Delivery; Microtask	Primary and secondary qualitative research
Yu et al. (2022)	China	Delivery	Interviews with 12 workers from 4 platforms

Source: Authors’ compilation from secondary literature. * signifies research that was carried out by the ILO.

## 3 Results and discussion: agency and resilience through resistance

Despite facing constraints imposed by algorithmic management, platform workers demonstrate agency through various forms of resistance in their pursuit of resilience. This section examines the strengths and limitations of both informal and formal resistance strategies employed across various platform sectors, such as online freelance and microtask platforms, taxi and delivery services, domestic and beauty services. From leveraging virtual private networks (VPNs) and online communities to organizing strikes and engaging in collective bargaining, workers utilize a range of tactics. These efforts aim to negotiate improved working conditions, challenge exploitative practices, and ultimately regain control over their livelihoods amidst algorithmic power, thereby fostering resilience. The analysis will highlight how these acts of resistance, often facilitated by digital connectivity, represent a dynamic interplay between technology as a tool of control and as a means of empowerment.

### 3.1 Navigating barriers to formal acts of resistance

In the face of limited structural and bargaining power, platform workers encounter significant obstacles in translating the collective solidarity developed in online groups into formalized acts of resistance typically facilitated by trade unions. While trade unions and worker associations engage in coordinated actions such as demonstrations, strikes, or collective logoffs, file strategic litigation, and conduct public advocacy through social media and regulatory channels (Hadwiger, 2022), their engagement remains relatively limited. This is particularly true for workers on online platforms operating in geographically dispersed, highly competitive environments (Rani et al., 2026). Analysis of the ILO survey of freelance workers, for instance, revealed that a vast majority (82.6 percent) had not sought assistance from labor unions, trade unions, professional associations or other organizations for their platform work. Only 12.9 percent reported referring to professional associations or organizations (ILO, 2021; Hadwiger, 2022).

Conversely, workers on location-based platforms operate within the same local labor markets, fostering face-to-face interactions and the identification of shared experiences. This proximity contributes to a higher incidence of visible collective action. The Leeds Index of Platform Labour Protest,^2^ for example, documented over 1,938 instances of platform worker protest across 57 countries between January 2017 and December 2022 in sectors such as ride-hailing, food delivery, courier, and grocery delivery. An earlier analysis, up to January 2020, found that digital strikes/logoffs (38.1 percent) or demonstrations (36 percent) were the most common forms of protest (Bessa et al., 2022). Despite this seemingly high number of protests, researchers noted that only a small percentage of workers actually participated in these formal acts of resistance (Bessa et al., 2022). For example, analysis of ILO cross-country survey results indicated that approximately 8.9 percent of taxi drivers and 3.4 percent of delivery workers had engaged in coordinated group actions, such as protests, demonstrations, or collective logoffs (Hadwiger, 2022; ILO, 2021).

Taxi and delivery workers who participated in these formal resistance activities were primarily motivated by a desire for increased pay, with secondary motives including resistance to algorithmic control, such as redefining bonus and incentive structures and preventing account deactivation (Hadwiger, 2022). However, approximately two-thirds of these workers reported that their objectives had not yet been realized. The limited success of these collective actions may stem from a lack of bargaining power and threats of deactivation or other punitive measures designed to discourage workers from participating (Hadwiger, 2022). This weaponization of algorithmic discipline reinforces platforms’ ability to dictate working conditions, thereby complicating formal acts of resistance.

**Figure 3:**
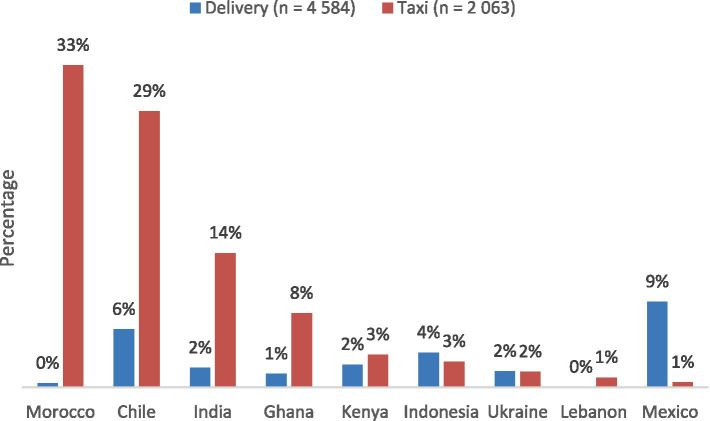
Share of app-based taxi and delivery drivers who have engaged in coordinated group actions. Source: ILO selected country surveys of taxi drivers and delivery workers (2019–2020). The figure illustrates the percentage of app-based taxi and delivery drivers who responded yes to, “While working as an app-based driver, have you participated in any coordinated group actions (protests, memorials, logging out of the app)?”.

In a few countries with strong institutional traditions, the literature has highlighted cases where workers have successfully organized collectively to establish works councils, leading to some evidence of resilience-building. However, workers in developing countries, in particular, face greater challenges in translating grievances into formal acts of resistance. Analysis of survey results revealed significant country-level variation: Chile (28.6 percent), India (13.9 percent), and Morrocco (33.3 percent) reported a significant proportion of taxi drivers who participated in coordinated group actions (e.g., demonstrations or collective logoffs), compared to Lebanon (1 percent), Mexico (0.5 percent), and Uganda (0 percent) (according to the analysis of more recent surveys) (Hadwiger, 2022; ILO, 2021, 2024b) (Figure 3). These country-level variations highlight the crucial role of industrial relations and cultural contexts in stimulating formal acts of resistance. While workers with access to traditional unions (most commonly in Europe) are more likely to hold union membership, workers in the Global South more frequently organize through informal groups to voice grievances, largely because platform companies are known to retaliate against workers involved in unionization, effectively discouraging union membership.

On the other hand, there is some evidence of collective action manifesting into resilience-building for workers on digital labor platforms in Europe, as highlighted in the literature. Collective organization among taxi and delivery workers has notably led to regulations and collective agreements addressing worker concerns related to algorithmic control, particularly regarding transparency and data protection. For instance, Spain, Law 9/2021 mandates that all workers, platform and non-platform alike, be informed about the “parameters, rules and instructions on which algorithms or artificial intelligence systems are based that affect decision-making that may have an impact on working conditions, access to and maintenance of employment, including profiling” (Hadwiger, 2022, p. 67). This regulation was further substantiated by a collective agreement between Just Eat Takeaway and trade unions, Unión General de Trabajadores (UGT) and Comisiones Obreras (CCOO), which includes data protection provision outlining workers’ right to information and establishing a joint “Algorithm Commission” (Hadwiger, 2022, p. 61). Similarly, in Denmark, a collective agreement is complemented by a sectoral agreement between Dansk Erhverv (Danish Chamber of Commerce) and 3F (the United Federation of Danish Workers) covering transport sector employees, including platform workers. This agreement explicitly prohibits the use of smartphones or other technological devices to monitor or track platform workers during their leisure time (Hadwiger, 2022). However, this right does not extend to workers driving company-owned vehicles under standard employment contracts.

Central to regulations governing algorithmic control on digital labor platforms in the European Union is the Platform Work Directive, formally adopted by the European Parliament and the Council of the European Union in October 2024. The Directive mitigates the risks of algorithmic management for platform workers by restricting certain automated practices, such as entirely automated deactivations, and by imposing higher transparency and explanation requirements. Going beyond the European Union’s General Data Protection Regulation (GDPR), the Directive addresses specific gaps related to algorithmic management in the workplace. For example, it defines platform obligations to disclose information to workers (Article 9), establishes a right to human oversight of automated decisions (Article 10), and provides clearly defined redress mechanisms for challenging unfair automated decisions (Article 13), among other provisions (Rainone and Aloisi, 2024; Aloisi et al., 2025). Unlike GDPR, the Platform Work Directive specifically targets digital labor platforms, tailoring its text to their unique operational context. This includes mandating greater transparency around automated management and decision-making systems and, in some cases, requiring the involvement of worker representatives in their oversight and evaluation (Article 12) (Aloisi et al., 2025).

Action research methods offer another institutional approach to resist digital labor platforms’ treatment of workers, notably through “naming and shaming” campaigns via social media and targeted local market initiatives. The Fairwork project exemplifies this by positioning researchers as “instigators of change,” evaluating and ranking platforms based on a predefined framework, assessing their adherence to minimum principles of fairness (Graham et al., 2020; Graham et al., 2025, p. 3). Principle 4, “Fair Management,” specifically assesses whether workers can appeal adverse decisions, such as automated deactivations, and whether algorithms are transparent and equitable (Graham et al., 2020). By publicly comparing platforms against these criteria and disseminating scorecards, the project encourages platforms to collaborate with research teams to improve their scores, thereby enhancing working conditions. As of early 2025, the program has resulted in over 320 pro-worker policy changes including improvements to appeal mechanisms and the institution of anti-discrimination policies (Graham et al., 2025). The Fairwork project thus illustrates how institutions can strengthen workers’ bargaining power by fostering collective action and driving positive competition among platforms.

### 3.2 Social media as the new “office watercooler”

Given the digitalized and geographically dispersed nature of work on digital labor platforms, workers frequently utilize social media groups and online forums to exchange knowledge and strategize ways to challenge algorithmic control. These online spaces are widely used by workers on online web-based and location-based platforms. A survey of microtask platforms workers, for example, found that nearly one-third of them leverage online forums to discuss problems and seek advice (Berg et al., 2018; Hadwiger, 2022). Analysis of survey data finds that freelance platform workers reported drawing on various sources, including YouTube (60.4 percent), blogs (36.1 percent), online courses or university programs (43 percent), forums and other online communities (48.3 percent), and topic-specific assistance platforms (38.5 percent) to build their knowledge (ILO, 2021; Hadwiger, 2022). For location-based platforms, a global survey revealed that 28.4 percent of app-based taxi drivers and 33.3 percent of app-based delivery workers are members of Facebook, WhatsApp, or other social media groups dedicated to platform work (ILO, 2021; Hadwiger, 2022). These members are generally active participants, with 80.1 percent of app-based taxi drivers and 76.8 percent of app-based delivery workers reporting exchanges multiple times per week in these groups. Similar results were observed in a survey of Ugandan platform workers, with 20 percent app-based taxi drivers and as high as 49 percent of delivery workers participating in such groups (ILO, 2024b). Finally, analysis of a survey of Kenyan domestic workers found 38 percent participated in social media groups (ILO, 2024a). These surveys highlight the critical role of such groups in addressing worker concerns and issues.

The goal of resilience-building strongly motivates workers to join social media groups. Analysis of the ILO surveys of taxi and delivery workers suggest that workers join with the hope or expectation of improving working conditions, and for a significant proportion, these expectations are realized, yielding substantial gains (84 percent among taxi workers and 74 percent among delivery workers in Uganda) (Hadwiger, 2022; ILO, 2021, 2024b). The benefits often come from information exchanges on local geographical conditions, including traffic updates, security alerts, and general news. Crucially, a common discussion topic is counteracting algorithmic control mechanisms, such as avoiding account deactivation or maximizing earnings under opaque platform rules. In the taxi and delivery sector, workers share their experiences on topics like identifying optimal routes, understanding bonus schemes, and accessing surge pricing (Hadwiger, 2022).

Table 2 presents selected responses from taxi and delivery workers, illustrating how social media groups foster community and serve as platforms for sharing strategies to maximize earnings, both within and outside the app. These findings are corroborated by several case studies showing delivery workers’ reliance on WhatsApp groups and WeChat groups in Europe and China, respectively, to learn strategies for adapting to changes to platforms’ rules affecting earnings and evaluation through private chats (Tassinari and Maccarrone, 2020; Ferrari and Graham, 2021; Yu et al., 2022).

**Table 2:** Qualitative responses to ILO survey question on how social media improves their working conditions.

Subject	Text (country)
App-based delivery drivers	
Access to work opportunities	“They give you information about the places with more movement” (Chile) “There are times when we get notified through the group if there is work in some areas” (Mexico) “I read tips about where is more work” (Mexico)
Knowledge exchange	“They help you when you enter, you know nothing” (Chile) “More friends, can exchange ideas, get other jobs” (Indonesia) “They gave me advice when I started to work as a courier, like in which hours is better to work” (Mexico)
How to maximize earnings	“I have read tips on how to earn more money” (Mexico) “They give information about bonuses and high demand areas” (Mexico) “Instructions for profitable order trends” (Lebanon)
Platform updates and failures	“They talk about failures in the app and recommendations are given” (Chile) “Informing us about updates in company to improve our performance” (Lebanon) “I read information about app failures and its geographical coverage” (Mexico) “We get information about which technical problems the app has” (Mexico) “Solve some application-specific problems” (Lebanon)
Community building	“There is a lot of help in solidarity” (Indonesia) “Support among couriers in case of emergencies” (Mexico) “They make the job more enjoyable because my courier friends are there” (Mexico)
Safety	“Security, there is monitoring between communities when driving” (Indonesia) “I have read advice about advice dangerous areas” (Mexico) “Warnings about in which areas there is a risk to suffer a crime” (Mexico)
Travel routes	“They tell me the route less dangerous and how I can get to the destination faster” (Mexico) “Delivery routes are published there” (Mexico) “They help me with doubts about how to get to some addresses” (Mexico)
App-based taxi drivers	
Access to work opportunities	“It helps us to find where booking is high in which area” (India) “We transfer rides to each other when one of us is not available” (Lebanon) “I see long trips that users request and we can make them outside of the platform” (Mexico) “It helps to know where areas the areas where I can find trips” (Mexico)
Knowledge exchange	“After getting low ratings on [redacted], about how to improve that” (India) “Exchange of information about what’s happening in the field” (Lebanon) “I benefit from other people’s experience” (Lebanon) “It’s school” (Morocco)
How to maximize earnings	“They chat about the fares and all and helps in fares” (India) “They give sometimes private rides in which we earn more” (India) “We warn where you can find dynamic pricings and I read news about the job” (Mexico)
Platform updates and failures	“A group of explanations of the application” (Morocco) “Get advice for solving work-related problems” (Morocco)
Community building	“We support each other and we are less stressed” (Chile) “Union support service does not help, so at least the guys help” (Ukraine)
Safety	“I have received help when I have problems. They have followed my trips sometimes. When I was taken to buy drug I sent a code to the group and they followed me by GPS” (Chile) “Security because we care for each other. Once I put an alert 37 (internal code) for a possible danger of robbery. I did not feel safe with the passenger and they monitored me through the Group, they were following me via Whatsapp in real time and through” (Chile) “I read advice about the road and I feel safer when I go to dangerous areas” (Mexico)
Travel routes	“We give assistance and information about the traffic and “insecurity” (Chile)” “I read advices about the road and I feel safer when I go to dangerous areas” (Mexico) “I read about where there are closed roads” (Mexico)

Source: ILO selected country surveys of taxi drivers and delivery workers (2019–2020).

Similarly, Tandon and Sekharan (2022) provide examples of worker resilience and resistance from Urban Company, an Indian beauty and home services platform, where workers use WhatsApp to adapt to changes in algorithmic design by posting marked screenshots with questions in groups. These posts included screenshots of earnings across geographic zones, enabling workers to reverse engineer how the algorithm determines earnings. The mobilization of collective knowledge thus proves to be a critical tool for identifying pathways to resilience in a context where algorithmic control is subject to unexpected changes.

**Figure 4:**
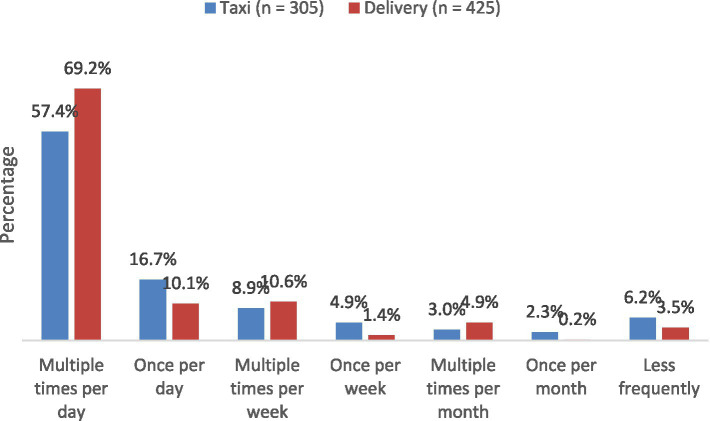
Frequency of work-related communication among app-based taxi and delivery workers who use social media to talk with other drivers. Source: ILO selected country surveys of taxi drivers and delivery workers (2019–2020) in Chile, Ghana, India, Indonesia, Kenya, Lebanon, Mexico, Morrocco, Ukraine. The figure illustrates the percentage of app-based taxi and delivery drivers who reported using social media to talk with other drivers about their work experience and responded yes to, “How often do you talk with other drivers about your work experience on social media?”.

Rather than solely relying on traditional unions, social media platforms and online groups serve as key mechanisms through which platform workers organize and strengthen collective activism. As evidenced in Table 2, these online groups foster a community where workers connect, offer mutual support, and share their experiences (Cini, 2022). These relationships that are developed are pervasive; a majority of workers who engage with peers through social media groups report daily communication about their work experiences according to analysis of survey data (Figure 4). This frequent exchange of shared experiences and grievances cultivates solidarity, laying the groundwork for more formalized acts of resistence. For example, Turkopticon, a browser plug-in designed for MTurk workers to exchange information and negotiate fairer work norms, demonstrates how a website initially intended for informal resistance can incubate collective formal resistance. Workers using the forum developed solidarity, which motivated them to engage in coordinated work refusals and brand shaming campaigns against Amazon, achieving some success (Altenried, 2020; Woodcock, 2021). Another MTurk engagement platform, Dynamo, organized two global campaigns to improve working conditions. The first targeted clients, guidelines for academic requestors, was developed outlining criteria for ‘good’ microtasks (Berg et al., 2018; Cini, 2022). The second initiative targeted the platform itself, encouraging workers to write personal letters to Amazon CEO Jeff Bezos (Salehi et al., 2015; Heiland, 2020; Cini, 2022). By attracting media attention, this campaign successfully changed the default payment method from check to direct deposit (Cini, 2022). These initiatives highlight the diverse and innovative strategies available to digitally dispersed workers, demonstrating how online spaces can facilitate formal acts of resistance, even in geographically fragmented labor environments.

### 3.3 Building resilience through informal acts of resistance

Informal acts of resistance are frequently adopted due to the structural barriers to collective organization faced by digital platform workers, which arise from a geographically dispersed workforce often lacking collective identity, as previously discussed. In female-dominated sectors, such as domestic work and beauty platforms, workers tend to assert their agency through subtle, informal resistance given the lack of formal alternatives. This tendency aligns with findings by Barua et al. (2016), who highlight that women are more inclined to participate in everyday forms of resistance to safeguard their well-being and livelihoods.

#### 3.3.1 Countering algorithmic access to work and allocation

Platform workers across sectors employ a variety of tools and techniques to resist algorithmic control over access to work and allocation – often manifested through task assignment and gamification. For workers on online freelance and microtask platforms, particularly in the global South, digital tools are leveraged to broaden access to higher paying tasks. VPNs serve as a key informal resistance tool, especially in bypassing geo-blocking restrictions. By using VPNs, workers can access higher-paying assignments typically reserved for those in the global North, effectively circumventing geographic wage disparities (ILO, 2021; Rani et al., 2023). Additionally, VPNs enable workers to create accounts, pass qualification exams, and secure tasks that better match their educational background and skill set. In some cases, these VPN-created accounts are sold through informal online groups (e.g., WhatsApp, Facebook) (Anwar and Graham, 2020; ILO, 2021; Rani et al., 2023). For workers without personal VPN access, purchasing accounts becomes their only option. However, risks arise when these accounts are deactivated, leaving the purchaser to bear the entire burden, rather than the seller (Rani et al., 2023). Location-based workers, conversely, employ different technologies to access a broader range of work, as demonstrated by Chen et al. (2025), who found that up to 40 percent of drivers use bot applications or register their vehicles on multiple devices to bypass algorithmic task assignments, allowing them to compare and select more favorable rides.

Rosenblat and Stark (2016, p. 3766) noted that certain platform-based drivers reported engaging in “trying to game the algorithm…” based on their practical experience with surge pricing’s “duration, reliability, and potential reward.” A compelling illustration of this phenomenon comes from Jakarta, where drivers were observed developing their own sophisticated counter-optimization strategies. These tactics, achieved through experimentation, peer consultation, and reverse engineering, is similar to the data science methodologies employed by platform algorithm designers (Qadri and D’Ignazio, 2022). By synthesizing information from multiple sources—including peer updates, weather forecasts, personal obligations, and digital tools such as Google Maps Timeline—drivers were able to critically analyze their work patterns, anticipate demand and order hotspots, and consequently adjust their positioning. These proactive strategies empowered drivers to exert significant agency over the platform, facilitating the optimization of their economic outcomes instead of passively allowing algorithmic direction (Qadri and D’Ignazio, 2022).

Workers also bypass algorithmic allocation by rejecting tasks that may yield low potential earnings relative to the time required. By rejecting (Tassinari and Maccarrone, 2020; Tandon and Sekharan, 2022), redistributing (Tandon and Sekharan, 2022) or threatening to cancel contracts (Anwar and Graham, 2020), workers challenge existing power dynamics regaining agency over their schedules and even negotiating higher earnings. However, this tactic was primarily used by workers with higher ratings, who were less concerned about the impact of declining tasks on their ratings in the beauty sector (Tandon and Sekharan, 2022). Their concerns were validated by respondents who reported persistent managerial pressure, including frequent calls urging them to accept tasks and, in some cases, the worker IDs were blocked. Given the limited bargaining power associated with informal acts of resistance, workers need to carefully calculate the risks associated with such actions. To avoid penalties, workers in domestic work and beauty platforms use social media to share work opportunities with others needing additional income, ensuring completion of the task without declining it themselves (Tandon and Sekharan, 2022). This example demonstrates the iterative process of resilience, where workers find ways to overcome platform policies designed to suppress resistance.

#### 3.3.2 Countering algorithmic monitoring

As previously described, platforms collect vast amounts of data on workers, including location, task completion times, and customer ratings. Workers on both location-based and online platforms circumvent algorithmic monitoring by connecting with clients off-platform. On online freelance and microtask platforms, workers particularly contact clients directly via social media platforms, such as LinkedIn, Facebook, and other sites. Although more time consuming, this allows freelance workers to negotiate higher wages and avoid algorithmic ratings and discipline (ILO, 2021). Similarly, domestic and beauty workers share contact information directly with clients who are satisfied with their services. By establishing relationships and convincing clients to exchange services outside the platform, workers bypass commission fees and algorithmic monitoring (Dhar and Thuppilikkat, 2022; Tandon and Sekharan, 2022). While building off-platform client relationships can be advantageous, it also poses significant risks, as platforms typically prohibit such interactions. An ILO survey of freelance platform workers found that 69 percent reported platform restrictions on working with clients off-platform (ILO, 2021). Workers not respecting the policy can face repercussions, including deactivation. Therefore, workers devise other creative ways to avoid algorithmic monitoring while remaining connected to the platform. For instance, some freelance and microtask workers use a second display screen, which allows them to work on other tasks during working hours without supervision (Anwar and Graham, 2020).

#### 3.3.3 Countering algorithmic evaluation

Since algorithmic evaluation influences pay, platform workers engage in informal acts of resistance to maximize earnings by preventing negative reviews. Some online freelance platform workers purchase positive reviews from clients who post fake jobs in exchange for good feedback and rankings or even share accounts with friends and family to maximize ratings (Anwar and Graham, 2020). To protect themselves from wage theft and harassment, workers have developed methods to withhold finished goods from clients until payments are made, in addition to leaving negative feedback and reviews on platforms with two-sided rating structures (Anwar and Graham, 2020). On location-based platforms, such as taxi and delivery services, workers engage in acts of resistance with varying risk levels. Among lower-risk actions, delivery workers in China, for example, work for several platforms concurrently, divert from platform-recommended routes, and use multiple phones to access multiple bonuses (Yu et al., 2022). Higher-risk acts of resistance include cooperating with restaurants to carry out fake orders, aiming to boost their number of orders and collecting delivery fees (Yu et al., 2022). This aligns with findings in the taxi sector, where drivers often reject trips if they anticipate that it will lead to negative ratings (e.g., pooled rides) to protect their ratings on the platform (Möhlmann and Zalmanson, 2017; Ferrari and Graham, 2021). However, these actions require careful consideration, as frequent cancellations or avoidance of undesirable tasks can trigger algorithmic penalties, including deactivation.

### 3.4 Platform responses to resistance

The earlier sections demonstrated methods through which workers exercise formal and informal resistance to improve their well-being. However, the process of developing resilience in the face of algorithmic control can be complicated by managerial responses. As platforms detect worker resistance strategies through algorithms designed to identify fraud or anomalies, they can respond by reinforcing algorithms with disciplinary measures or disincentives, further embedding algorithmic control into work processes (Dasgupta et al., 2025b). While literature on managerial responses to resistance is limited, case study evidence from the domestic work and beauty sector in India provides insights into how platforms react to such acts of resistance.

Dhar and Thuppilikkat (2022) and Tandon and Sekharan (2022) examine several protest tactics undertaken by women on the Urban Company platform in New Delhi, India aimed at eliciting systemic policy changes. Growing pressure stemmed, in part, from policy changes within the application that expanded algorithmic control, further restricting the flexibility that had initially attracted workers to the platform (Dhar and Thuppilikkat, 2022). However, collective action was ultimately triggered in October 2021 when a female worker attempted suicide after her ID was arbitrarily blocked by an algorithmic decision. This incident’s video went viral in WhatsApp groups, leading women to participate in a series of protests (Dhar and Thuppilikkat, 2022).

According to Tandon and Sekharan (2022), some workers initially acted privately by filing notices against the platform, complaining about the introduction of algorithmic control practices, such as ID blocking, earnings deductions, and penalization for customer-led cancellations. However, lawyers informed the women workers that filing legal notices required proof of employment, yet the only documentation available were standard terms and conditions. This example illustrates how the non-traditional employment relationships imposed by platforms limit workers’ structural power.

In response to early protests, Urban Company representatives initially met with a delegation of 10–15 workers in October 2021, but negotiations were unfruitful. The Company’s stance shifted when workers began staging public demonstrations—including sit-ins, road blockages, and protest marches—which raised the visibility of workers’ experiences and threatened the platform’s reputation (Dhar and Thuppilikkat, 2022; Tandon and Sekharan, 2022). Following the protests, workers were brought back to the bargaining table, where 12 of their 13 demands were accepted. Their victories included capping commission rates, establishing an SOS helpline, and allowing penalty-free cancellations, among other key concessions. On the one hand, the success of these formal resistance efforts underscores the power of public pressure in safeguarding the company’s reputation. On the other hand, these events sparked backlash from Urban Company against involved workers. Some received legal notices for destruction of property. In addition, the platform employed intimidation tactics such as ‘shadow blocking’—a covert disciplinary measure where workers’ accounts remain active on the application but do not receive any work (Tandon and Sekharan, 2022, p. 701). In other cases, managers infiltrated WhatsApp groups to identify members for blocking or to penalize them. These examples illustrate a cycle where workers who organize to challenge algorithmic control are met with intensified algorithmic restrictions, further complicating their ability to mobilize and resist.

## 4 Conclusion

This article has examined how platform workers deploy diverse acts of resistance to develop resilience strategies against the pressures of algorithmic control, reframing resistance not merely as an outcome but as a crucial component within the dynamic process of adapting to the changing platform environment. The analysis reveals a key paradox: algorithms, the very source of disruption and precarity requiring resilience, simultaneously serve as tools through which workers organize, connect, and enact resistance. This digital mobilization, facilitated by social media’s low barrier to entry, is particularly critical given the geographically dispersed and often precarious nature of platform work.

The structural context of platform work, often characterized by self-employment and a lack of traditional employer-employee relationships, presents significant challenges to unionization. Formal collective bargaining, which is vital for worker protection in traditional employment, has gained prominence in jurisdictions with established institutional bargaining mechanisms but remains less prevalent, especially in developing countries. ILO surveys consistently demonstrate low union membership rates across various platform sectors, especially in developing countries, highlighting the difficulty of organizing a workforce dispersed across geographical boundaries and often operating under precarious contractual arrangements. This absence of strong, formalized union structures makes informal acts of resistance, such as sharing tips online, bypassing geographical restrictions using VPNs, negotiating wages off-platform, and strategically manipulating algorithms, essential for workers to adapt to and contest algorithmic control over work organization. While often individually enacted, these informal tactics can contribute to a broader sense of collective agency and lay the groundwork for more formalized resistance efforts.

The findings also demonstrate that platforms often introduce intensified algorithmic control mechanisms, such as ratings and task allocation algorithms, primarily after achieving significant market dominance and lock-in of workers in the platforms. This suggests that these control mechanisms, while presented as essential for operational efficiency and platform governance, are primarily geared towards maximizing platform profits and consolidating power over workers, rather than being intrinsic to core platform functionality. The case of the Finnish platform, Wolt, successfully operating without ratings underscores this point. Moreover, in scenarios of increased labor supply and consequently depressed wages, collective worker action, even in the absence of traditional unions, can still leverage bargaining power to negotiate improved terms and challenge exploitative practices.

Platform workers increasingly have a diverse array of tools to exercise their agency, which are shaped by the digitally mediated context in which they operate in. As platforms deploy new mechanisms of algorithmic control, workers continuously adapt and develop new and innovative resistance strategies to bolster their resilience. However, the process of resilience building is often more complex and nuanced. As the example of Urban Company demonstrates, workers’ resistance is often met by intensified or targeted forms of algorithmic control from the platforms. The pattern of action followed by reaction may represent a continuous game of cat and mouse, which, on the one hand, has the potential to discourage workers from exercising resilience and further consolidates platform power. On the other hand, this iterative approach to resilience-building can further motivate workers by fostering collective organization and learning through the process of adapting, resisting and negotiating the features of algorithmic management. Such opportunities for building collective solidarity are crucial for the development of structural power across sectors.

However, our analysis is not without limitations. The dynamic, context-dependent, and multi-faceted nature of resilience is inevitably simplified within our framework. Factors such as individual adaptability, the broader economic climate, existing labor market institutions, and the specific regulatory environment significantly influence the process of resilience-building. Resistance, while central to our argument, represents only one facet of this complex process. Future research should further explore these nuanced interplays, investigating the long-term effectiveness of various resistance strategies, the evolving relationship between algorithmic control and worker agency, and the potential for hybrid forms of worker organization to emerge in the platform economy.

## Data Availability

The data analyzed in this study is subject to the following licenses/restrictions: the ILO survey datasets that were used for the analysis of ILO-affiliated reports are not yet publicly available. Requests to access these datasets should be directed to williams@ilo.org.

## Footnotes

1 The platform management methods (PMM) outlined in Moore and Joyce (2020) and Joyce and Stuart (2021) include: algorithmic allocation of work; digital tracking and monitoring of workers; integration of customer ratings into performance management systems; setting of prices for services provided and rates of pay for work conducted; extraction of commission on every transaction; engagement of workers on self-employed or independent contractor status; and legal and regulatory arbitrage concerning worker status and service provision.

2
https://leeds-index.co.uk/
